# Hamiltonian path analysis of viral genomes

**DOI:** 10.1038/s41467-018-03713-y

**Published:** 2018-05-22

**Authors:** Reidun Twarock, German Leonov, Peter G. Stockley

**Affiliations:** 1 York Cross-disciplinary Centre for Systems Analysis, University of York, York, YO10 5DD UK; 2 Departments of Mathematics and Biology, University of York, York, YO10 5DD UK; 3 Astbury Centre for Structural Molecular Biology, University of Leeds, Leeds, LS2 9JT UK

## Introduction

Cryo-electron microscopy (EM) is undergoing a revolution, enabling the study of viral pathogens in unprecedented detail. The asymmetric EM reconstruction of bacteriophage MS2 at medium resolution (8.7 Å) by Koning et al.^1^, and the subsequent reconstruction at even higher resolution (3.6 Å) by Dai et al.^2^ revealed the structures of both the protein shell and the asymmetric genomic RNA and the unique maturation protein (A). It is the start of a wave of such structural data for viruses, and calls for the development of new analytical tools to describe the results. One approach is Hamiltonian path analysis (HPA) that we introduced to describe repeated, sequence-specific contacts between the MS2 genome and its protein shell^3^. Here, we describe how HPA is consistent with the new structures and, in turn, how it extends our understanding beyond the structural data alone.

Koning et al.’s and Dai et al.’s reconstructions of MS2 reveal multiple contacts between genomic RNA and the viral capsid. These mimick the contacts seen by crystallography of virus-like particles carrying multiple copies of the high-affinity RNA packaging signal in this virus, the translational repressor (TR) that functions also as assembly initiation signal^4^. TR occurs only once in the MS2 genome, but insights into the roles played by RNA-coat protein (CP) contacts during assembly^5^ together with normal mode analysis^6^ and structural studies suggest that many different stem loops (SLs) in the viral genome should be able to bind CP and thus promote virion formation, i.e., act as RNA packaging signals (PSs). HPA has enabled us to identify such sites within the MS2 genome^7^, which is important, because it underlies the development of a new paradigm for ssRNA virus assembly based on multiple PSs, that seems to occur very widely in nature^8–11^.

**Fig. 1:**
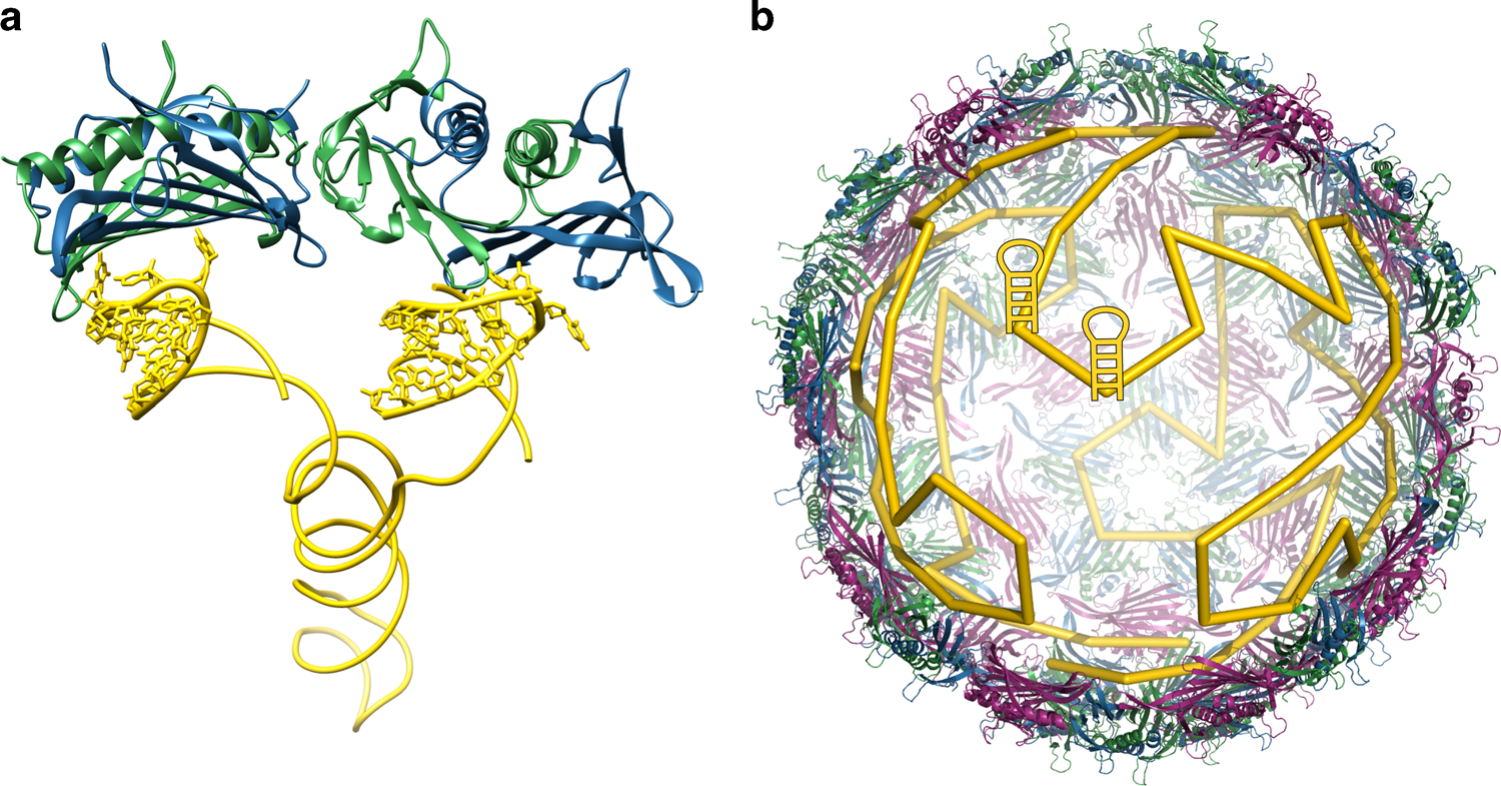
Hamiltonian path representation of MS2 genome organisation. **a** Two consecutive PS sites in the linear genomic sequence are mapped on adjacent binding sites in the inner capsid surface. The stem loops (PDB ID: 5TC1) and the backbone connecting them were reported in ref. ^2^. The coat protein shell is shown as ribbons based on the icosahedrally averaged X-ray structure (PDB ID: 1ZDH^12^). **b** These neighbouring PS contacts are represented by HPA as a short segment of an inscribed path connecting binding sites in the capsid interior, irrespective of their relative distance in the linear genomic RNA and the tertiary structure of the RNA fragment connecting them

HPA is a mathematical abstraction of virus assembly pathways, simultaneously encoding the order in which capsomers are recruited to the growing capsid shell along different assembly pathways. It captures geometric constraints on PS positions in the linear genomic sequence that arise from the relative positions of the RNA-CP-binding sites in the inner capsid surface (Fig. 1). SLs in close proximity in the linear genomic sequence acting as PSs must occupy proximal CP-binding sites on the inner capsid surface. SLs distal in the genomic sequence can also potentially be neighbours on the capsid surface, provided that the RNA segment between them occupies the capsid interior so as to bring them into proximity. Such constraint sets are akin to those of a large Sudoku puzzle, and HPA collectively tests them against experimental data. In particular, in HPA a polyhedron is used to represent all possible connections between neighbouring CP-PS contacts, with vertices at the binding sites and edges representing these (possible) connections. Each CP-PS contact is unique, i.e., can only occur once. We therefore represent the order in which these contacts are formed pictorially as an inscribed self-avoiding path on the polyhedron (see Fig. 1 bottom for an example of such a path). We stress that, in mathematics terms, this path is only 'topologically equivalent' to the more complicated 3D path taken by the RNA, meaning that the connectivity between binding sites with reference to the linear genomic sequence is the same in both cases (see Fig. 1 top, illustrating how a genomic fragment containing three PSs could map into 3D biology; PDB ID: 1ZDH^12^). We explicitly assume that the RNA genome is highly branched, since we predict these contacts to be with SLs located within the MS2 genome^7^, as seen in the new reconstruction. The Hamiltonian path concept is thus an abstraction of discrete 3D contact sites into a linear path that should be understood in the same spirit as the simple lines between atoms in molecular structures are shorthand for the much more complex electronic arrangements of covalent bonds. We note that HPA does not require all possible RNA-CP sites to be occupied. Indeed, testing all possible SLs in the ensemble of putative PS candidates against all complete Hamiltonian paths would be a complex task, and indeed is not always possible as different interaction patterns can occur in the vicinity of asymmetric features such as the A-protein^13^.

**Fig. 2:**
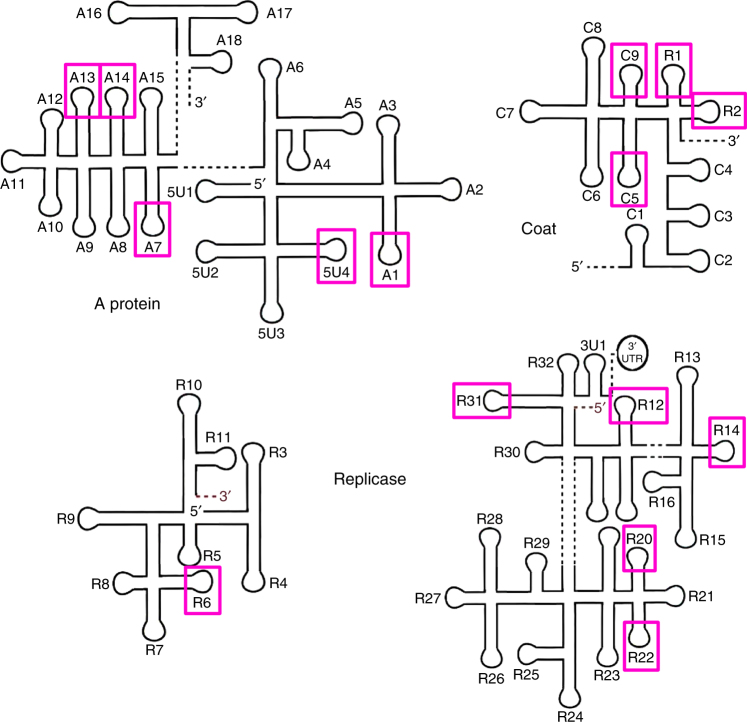
PSs in the MS2 genome predicted by HPA analysis and confirmed by cryo-EM. The secondary structure of the MS2 genome is shown as a series of stem-loops (SLs), with the 15 SLs seen in the TR binding sites of the phage coat protein dimers in the asymmetric cryo-EM reconstruction^2^, and predicted to be PSs via HPA (Dykeman et al^7^), indicated by magenta boxes. Other SLs in the genome share characteristic MS2 coat protein recognition motifs with these 15 PSs, and were also proposed to be PSs. It is possible that at least some of these also contact coat protein dimers during PS-mediated assembly, but disengage thereafter and thus are not visible in the virion. Genomic SLs are numbered increasing in the 5′-to-3′ direction, preceded by their genetic locus, i.e. 5′UTR (untranslated region) (5U#), A protein (A#), CP (C#), replicase (R#) and 3′UTR (3U#).

An application of HPA to MS2^7^ has revealed that binding sites are differentially constrained across the capsid surface, implying that some (highly constrained) ones are likely to be present in almost every particle, while others (mostly low affinity ones) are more likely to vary across different particles, thus predicting that the RNA conformation in contact with the protein shell will exhibit some similar structural characteristics in every viral particle. This astonishing conclusion is consistent with both Koning et al.’s and Dai et al.’s reconstructions of MS2, identifying a core set of contacts that are present in every particle. Dai et al. explicitly identified 15 RNA SLs in contact with CP and one in contact with the A-protein. Our HPA in Dykeman et al.^7^ has predicted all of these 15 RNA-CP contacts as shown in Fig. 2. The additional PSs that were also identified by HPA are predominantly of lower affinity to CP, and are therefore not expected to be present in every particle. The results of HPA are also in excellent agreement with the RNA-CP-binding sites identified via cross-linking immunoprecipitation (CLIP) experiments^14^

The HPA has been key in identifying the nature of the RNA-CP contacts in MS2, and thus is fundamental to our understanding of the virion structure. It has played a central role in establishing the packaging signal hypothesis^3,9–11,15–18^, and in understanding how PSs cooperatively promote efficient virus assembly^16^. This suggests that HPA should be useful also for the study of the many other viruses, whose complete structures are likely to emerge in the near future from modern EM studies. Such structures will also highlight those viruses that exploit the multiple RNA packaging signal-mediated assembly mechanism.

## References

[1] Koning RI (2016). Asymmetric cryo-EM reconstruction of phage MS2 reveals genome structure in situ. Nat. Commun..

[2] Dai X (2017). In situ structures of the genome and genome-delivery apparatus in a single-stranded RNA virus. Nature.

[3] Dykeman EC (2011). Simple rules for efficient assembly predict the layout of a packaged viral RNA. J. Mol. Biol..

[4] Valegård K, Murray JB, Stockley PG, Stonehouse NJ, Liljas L (1994). Crystal structure of an RNA bacteriophage coat protein-operator complex. Nature.

[5] Stockley PG (2007). A simple, RNA-mediated allosteric switch controls the pathway to formation of a T = 3 viral capsid. J. Mol. Biol..

[6] Dykeman EC, Stockley PG, Twarock R (2010). Dynamic allostery controls coat protein conformer switching during MS2 phage assembly. J. Mol. Biol..

[7] Dykeman EC, Stockley PG, Twarock R (2013). Packaging signals in two single-stranded RNA viruses imply a conserved assembly mechanism and geometry of the packaged genome. J. Mol. Biol..

[8] Prevelige P (2016). Follow the yellow brick road: a paradigm shift in virus assembly. J. Mol. Biol..

[9] Shakeel S (2017). Genomic RNA folding mediates assembly of human parechovirus. Nat. Commun..

[10] Patel N (2017). HBV RNA pre-genome encodes specific motifs that mediate interactions with the viral core protein that promote nucleocapsid assembly. Nat. Microbiol..

[11] Stewart H (2016). Identification of novel RNA secondary structures within the hepatits C virus genome reveals a cooperative involvement in genome packaging. Sci. Rep..

[12] Valegård K (1997). The three-dimensional structures of two complexes between recombinant MS2 capsids and RNA operator fragments reveal sequence-specific protein-RNA interactions. J. Mol. Biol..

[13] Geraets JA (2015). Asymmetric genome organization in an RNA virus revealed via graph-theoretical analysis of tomographic data. PLoS Comp. Biol..

[14] Rolfsson O (2016). Direct evidence for packaging signal-mediated assembly of bacteriophage MS2. J. Mol. Biol..

[15] Borodavka A, Tuma R, Stockley PG (2012). Evidence that viral RNAs have evolved for efficient, two-stage packaging. PNAS.

[16] Dykeman EC, Stockley PG, Twarock R (2014). Solving a levinthal’s paradox for virus assembly suggests a novel anti-viral therapy. PNAS.

[17] Patel N (2015). Revealing the density of encoded functions in a viral RNA. PNAS.

[18] Patel N (2017). Rewriting nature’s assembly manual for a ssRNA virus. PNAS.

